# Comparison between methods for determining the effective vertical yield stress of intermediate fine-grained soils

**DOI:** 10.1038/s41598-023-50026-2

**Published:** 2024-01-02

**Authors:** Bartłomiej Szczepan Olek, Magdalena Moskal

**Affiliations:** Faculty of Civil Engineering, Cracow University of Technology, Cracow, Poland

**Keywords:** Civil engineering, Materials science

## Abstract

Effective vertical yield stress (σ′_xy_) is essential in accurately describing fine-grained soils’ mechanical properties and their behaviour under load over time. It helps assess settlements and stress history. In most constitutive models, this parameter indicates changes in the soil behaviour due to the development of recoverable and irrecoverable strains resulting from loading. The results of oedometric compression tests for 25 soil samples with a wide range of plasticity parameters were used for the investigation. The intermediate fine-grained soils comprised different proportions of clayey, silty and sandy fractions. An in-depth, two-staged statistical analysis was carried out to compare twelve methods of determining effective vertical yield stress, namely: Casagrande (CM), Pacheco Silva (PSM), Butterfield (BTM), Oikawa (OIM), Onitsuka (ONM), Boone (BM), Becker (WM), Morin (WPUVSM) Wang & Frost (DSEM), Tavenas (SEM), Senol (SLSEM), and Janbu (JM). It aimed to check the association of these methods and the consistency of the obtained results. Based on the difference analysis, the methods originated in the work approach (i.e. WM, WPUVSM, DSEM) and CM gave comparable σ′_xy_ values. The methods utilised bi-logarithmic plots (i.e. BTM, OIM, ONM) received slightly greater or lesser σ′_xy_ values than BM and JM. The remaining methods were characterised by medium to the high variability and were sensitive to even the slightest disturbances resulting from the procedure of determining σ′_xy_.

## Introduction

In the field of geotechnical and geological engineering, the behaviour of soil is commonly assessed by means of laboratory investigation combined with constitutive modelling. The predictions for the soil response under any arbitrary stress path require an appropriate model's mathematical formulation. The elasto-plastic theory assumes that the material response is partly reversible and partly irreversible in response to applied forces. This implies the possibility of strains decomposing into recoverable elastic strains (ε_e_) and irrecoverable plastic strains (ε_p_). The transition between the elastic and plastic region is called yielding^1^, whereby this term should be regarded as a gradual transition from the elastic region to the plastic region rather than yielding occurring as a single point between the regions^2^.

The inviscid (rate-independent) elastic and time-dependent plastic behaviour can be described using elastic-viscoplastic models, which belong to the family of general stress–strain-time models^3^. Sekiguchi^4^ categorized the elastic-viscoplastic models into two main classes. The first one constitutes models based on the concept of overstress and corresponding Perzyna's overstress theory. The second covers elastic-viscoplastic models based on the concept of a nonstationary flow surface^5^. By using the concept of overstress, the decomposition of strains can be extended, taking into account the term of time, as the elastic strains are time-independent, whereas the inelastic strains are time-dependent. However, in such an understanding, the mathematical formulation of the model is based on strain rates so that the total strain rate is additively composed of the elastic and viscoplastic strain rates. It is generally accepted that the elastic strain rate is assumed to obey the generalized Hooke’s law. In contrast, the viscoplastic strain-rate is expected to follow the non-associated flow rule. The main difference between the overstress theory and general elastoplasticity is the definition of inelastic strains. In the overstress models, inelastic strains are not related to the stress history but to the current stress state only, while in elastoplasticity, inelastic strains are related to the stress rate.

Despite these theoretical frameworks, from the practical point of view the transition from elastic to plastic soil behaviour is usually interpreted as a clear change in the stress–strain response. By convention the effective vertical yield stress (σ′_xy_) also known as ‘pre-consolidation stress (σ′_p_) is used to characterize the compressibility of soil. Casagrande^6^ defined preconsolidation pressure as the maximum past vertical effective stress applied to the soil and correlated it to the change in a slope of void ratio versus logarithm vertical effective stress curve^7^. The term ‘pre-consolidation stress’ may be inappropriate because it shows that only one mechanical factor causes the change in soil behaviour during loading. The visible change in soil response may be a result of many factors, including not only the past depositional and stress history but also changes in water content, chemical-physical reactions (weathering, cementation, recrystallization of minerals, ion exchange, modification of the adsorptive water layer or intermolecular attraction forces of clay minerals), cold welding, bonding due to long-term secondary compression, ageing, and other diagenetic factors^8–10^. Moreover, some observations indicated that soil might show a so-called preconsolidation pressure much greater than the existing effective stress without evident erosion in its geological history ^11^, or the pressure may decrease with increased soil depth^12^. Therefore, following Gao^13^, the term ‘vertical effective yield stress (σ′_xy_) will be used in this work as more fundamentally correct. The vertical effective yield stress points to the stress level corresponding to the onset of an irreversible mechanical behaviour of soil, and hence is useful for analysing and predicting settlement behavior, overconsolidation ratio (OCR), stress history, and short-term stability problems in fine-grained soils^14–18^. When σ′_xy_ is greater than the current effective stress, the stress–strain relation exhibits softening behaviour^19^, and the soil is in an over-consolidated state. In turn, strain hardening occurs when σ′_xy_ is equal to the current effective stress, and the soil in this case is in a normally-consolidated state.

The present work deals with the issue of determining the vertical effective yield stress in the intermediate soils consisting of different proportions of clay, silt and sand fractions using available methods. An in-depth, two-staged statistical analysis was carried out to compare twelve methods. Its purpose was to determine the similarity of these methods and the consistency of the obtained results. The main goal of the research was to investigate whether the values of σ′_xy_ determined by different methods are repeatable (or similar) and evaluate the rank of discrepancies between the obtained values. The intention was to indicate practical approaches that can be used interchangeably and are the least ambiguous. In this respect, the soils collected from the selected area located in Poland were studied through incremental oedometer tests.

## Overview of existing methods for determining σ′_xy_

Over the past decades, various empirical methods for determining the value of the vertical effective yield stress (σ′_xy_) have been developed. The most popular methods include the graphical interpretation of the oedometric compression curve described by the relationship between void ratio and effective vertical stress and their different modes. This paper focuses on twelve different methods, namely: Casagrande (CM), Pacheco Silva (PSM), Butterfield (BTM), Oikawa (OIM), Onitsuka (ONM), Boone (BM), Becker (WM), Morin (WPUVSM) Wang & Frost (DSEM), Tavenas (SEM), Senol (SLSEM), and Janbu (JM). Based on previous observations, the conclusion has been made that all these methods might give different results when considering a particular compression curve. In general, this is related to the subjective assessment of the inflection point of the soil compression curve or the influence of the scale of the graph. The quality of the collected and tested soil samples is also essential in correctly determining the preconsolidation pressure. To minimize subjectivity in determining the compression parameters, the authors used the open-source software *pySigmaP*^20^.

### Semi-logarithmic approach

**Figure 1:**
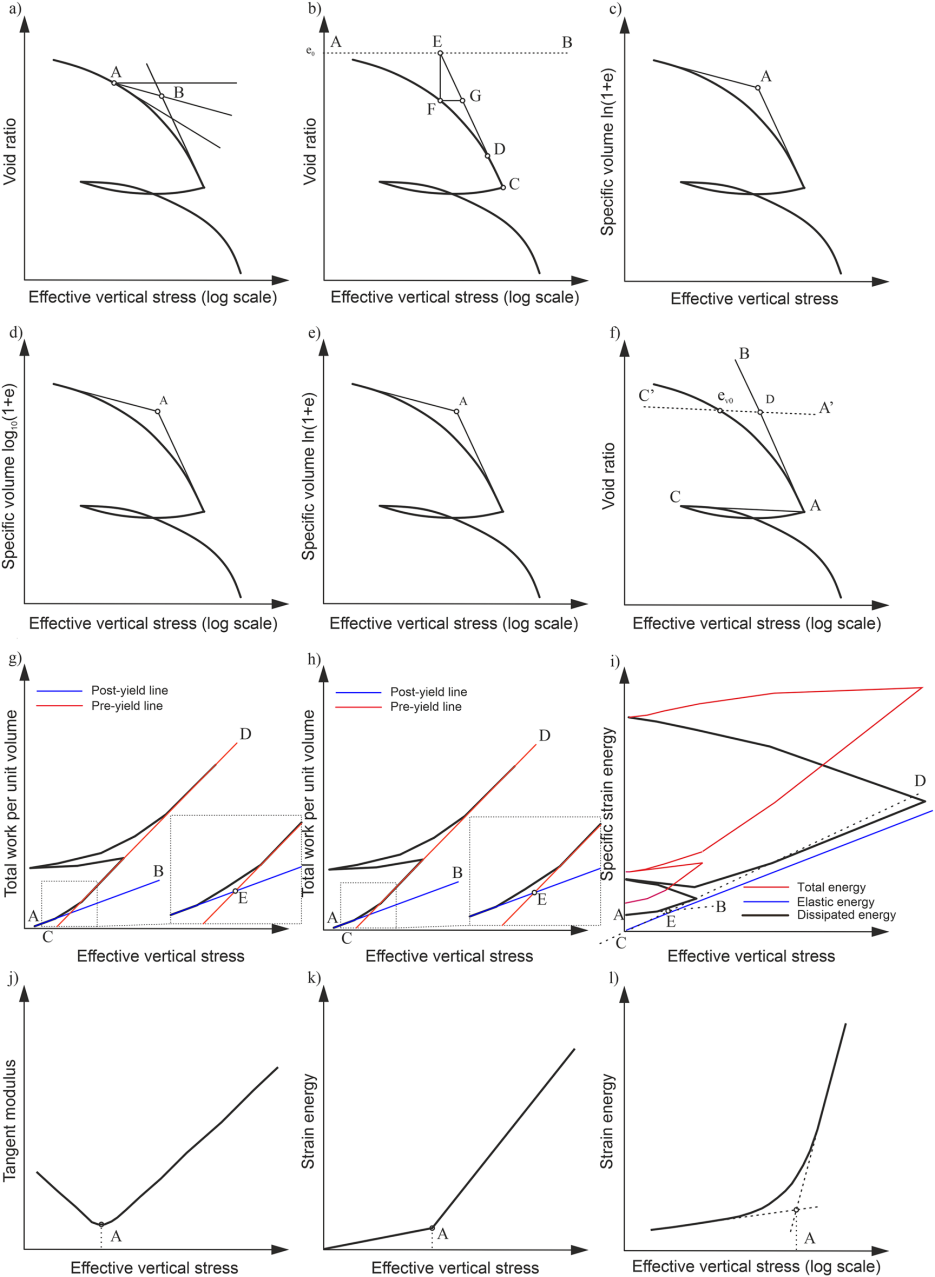
Schematic interpretation diagrams for estimating the effective vertical yield stress using: (**a**) CM, (**b**) PSM, (**c**) BTM, (**d**) OIM, (**e**) ONM, (**f**) BM, (**g**) WM, (**h**) WPUVSM, (**i**) DSEM, (**j**) SEM, (**k**) SLSEM, (**l**) JM.

Arthur Casagrande^21^ was probably the first researcher who used the semi-logarithmic approach to estimate σ′_xy_. Although many researchers judged his pioneering method as inaccurate^22^, it is still remains a standard for comparison to other methods^23^ and recommended in widely used standards (i.e. ISO 17,892-5, ASTM D2435). To determine σ′_xy_, the inventor used a semi-logarithmic plot of the void ratio e versus the effective vertical stress. The methodology for determining σ′_xy_ using the Casagrande method (CM) is illustrated in Fig. 1a. The graphical construction involves determining the minimum radius of curvature point on the compression curve and drawing several additional lines. Certain of authors showed that the selection of this point strongly depends on sample disturbance and water content of soil (e.g.^24–27^). It should be mentioned that semi-logarithmic approach has become the target of further improvements and refinements. For example Dawidowski and Koolen^28^ developed computerized version of CM. Moreover, Peck et al.^29^, for sensitive soils, simplified the procedure, limiting themselves to determining the point of inflection on the curve and the point of intersection of the tangent line at this point with the so-called initial void ratio line.

In turn, Pacheco Silva^22^ introduced the method called Pacheco Silva method (PSM), accounting for independence from the drawing scale^30^. The method utilised the initial void ratio line and graphical construction to determine σ′_xy_, as shown in Fig. 1b. Grozic et al.^31^ have demonstrated that the determined σ′_xy_ by this method depends on locating the tangent to the virgin consolidation line.

Further, Boone^7^ noted that the CM does not always give accurate results, especially for soils that do not show a well-defined change in curvature in the graph. He also recognized that the derived value of the initial void ratio e_0_ from the oedometer is not a favourable approach due to the sample's disturbance issue. On the basis of the above, the so-called Bonne method (BM) has been proposed. The methodology for graphically determining σ′_xy_ using BM is illustrated in Fig. 1f. The method requires establishing the in-situ void ratio e_v0_ corresponding to the in-situ effective vertical stress σ′_v0_ (directly using load increment corresponding to σ’_v0_ or using the interpolation). The reader is referred to the original paper for a detailed empirical investigation and graphical procedure^7^.

### Bi-logarithmic approach

Butterfield^32^ has proposed an alternate approach for describing soil volume change with variations in effective vertical stress. It has been considered to plot ln(1 + e) versus ln mean effective stress (p′). The so-called bi-logarithmic method (BLM) used to characterise soil compressibility implies some advantages over the classic logarithmic plot in that it might be applied to large-strain deformation and clarifies the influence of the unloading stress ratio on reloading response and compression parameter values^33^. Moreover, the bi-logarithmic relationship might show improved linearity with experimental data for unloading and reloading sections of the compression curve.

In the case of determining σ′_xy_, the linear portions at both ends of the original compressibility curve are extended. The point of their intersection (see point A) determines the sought value of σ′_xy_. The bi-logarithmic nature of the compression behaviour has been reported for many different soils^9,18,34–41^. The bi-logarithmic approach includes several methods for which the procedure for determining σ′_xy_ is the same, and only the form of mathematical relationship used differs. For instance, Onitsuka et al.^39^ supported the use of the ln(1 + e) – log p′ plot, while Oikawa^42^, Jose et al.^23^ and Sridharan et al.^9^ preferred the log (1 + e) − log p′ plot. From the methodological aspect, the proposal of Onitsuka et al.^39^ has strong theoretical background because it might be related to the work approach ^43^. The methodology for determining σ′_xy_ using the methods of Butterfield (BTM), Oikawa (OIM), and Onitsuka et al. (ONM) methods are illustrated in Fig. 1c,d,e.

### Work approach

Another interpretation approach has been proposed by Becker et al.^44^. The idea behind it was based on the definition of work (W) (total strain energy or energy per unit volume). Here this method is called as work method (WM). The authors defined incremental work (cumulative total strain energy) using the following formula:

1 $${\Delta W}_{oed}=E=\left(\frac{{\sigma {\prime}}_{i+1}+{\sigma {\prime}}_{i}}{2}\right)\bullet ({\varepsilon }_{i+1}-{\varepsilon }_{i})$$

where:

${\sigma {\prime}}_{i+1}+{\sigma {\prime}}_{i}$—effective stresses at the end of load increments i + 1 and I, ${\varepsilon }_{i+1},{\varepsilon }_{i}$—natural strain at the end of load increment i + 1 and given i-th.

Principles of WM are shown in Fig. 1g. The plot in an arithmetic scale in which the relationship between work per unit volume (W) and consolidation pressure (p) is expressed in bi-linear lines has been used to determine σ′_xy_. The intersection of the so-called preyield (see AB line) and post-yield (see CD line) lines when the work incremental rate ΔW/Δp increases significantly has been interpreted as σ′_xy_.

Morin^45^ undermined the original way of computing the work per unit volume using cumulating incremental work values per unit volume. As an alternative, the work per unit volume of solids had been recommended (say work per unit volume of solids method (WPUVSM)). Hence, the two methods differ only in the mathematical formulation of work per unit volume for a given load increment, while the procedure for determining the σ′_xy_ is the same.

More recently, Wang and Frost^46^ have improved the original WM. They considered the consolidation process irreversible, in which most of the work done during it is dissipated. Based on this foundation, the dissipated strain energy method (DSEM) was introduced to describe pre and post-yield behaviour based on plastic deformations. The schematic of DSEM in the specific strain energy (SSE) versus effective vertical stress space is shown in Fig. 1h. The determination of the σ′_xy_ does not differ from other methods based on the work approach except that it applies to the dissipated energy.

### Other methods

So far, alternatively, to the semi-logarithmic, bi-logarithmic and work-based methods, some other methods of determining σ′_xy_ have been developed. Likely to the methods mentioned in the previous section, they are mainly based on observing strains exhibited by the soil material during oedometric tests. Certain exceptions are methods developed based on the time resistance approach^47^ and the analysis of the variation of the tangent modulus (M = Δσ/Δε) as a function of stress. Unfortunately, the original description of the Janbu procedure (say Janbu method (JM)) is very vague. In some cases, the authors indicated the use of the relationship between M and consolidation pressure on a linear scale (see Fig. 1j), and in others, such as in the case of Jacobsen^48^, the relationship between deformation and consolidation pressure on a semi-logarithmic scale. In the first case, σ′_xy_ was obtained by locating a downward concavity on the graph. In the second, the experimental data was approximated by two straight lines, and their intersection pointed to the σ′_xy_, similar to bi-logarithmic methods. In addition, two variants of the Janbu approach can be found in the literature (i.e^49,50^). Other examples are methods designed for CRS studies^51,52^ or based on shear wave velocity at small strains^53^.

Alternatively, researchers have developed methods based on the strain energy concept (SE = σ′ΔH/H). In this connection, Tavenas et al.^19^ considered the intersection of two inclined lines on the plot of SE versus consolidation pressure in a linear scale as a σ′_xy_ (see Fig. 1k). Hereafter, in this paper, this method is denoted as the strain energy method (SEM). Moreover, Senol and Saglamer^54^ studied the semi-logarithmic relationship between SE and consolidation pressure (see Fig. 1l). Thus, this method is denoted as the semi-logarithmic strain energy method (SLSEM).

To sum up, the past studies have all highlighted issues related to the practicalities of the graphical interpretation of consolidation test data. For instance, Schmertmann^27^, Brumund et al.^24^, and Holtz and Kovacs^26^ pointed out that the semi-logarithmic approach applied to disturbed soils may be inaccurate due to the difficulty in determining the inflection point on the compression curve. Grozic et al.^31,43^, Clementino^30^ and Boone^7^ provided detailed comparisons between various methods. The main conclusion drawn from their considerations is that most of them are burdened with uncertainty and, in many cases, challenging and ambiguous to apply. To put it briefly, the technicalities of most of the methods are concerned with defining the tangent or "best-fit" lines and reading of yield point. The main error sources come from subjectivity and the plot type used for interpreting test results (i.e. scale effects).

## Experimental program

### Test materials

**Figure 2:**
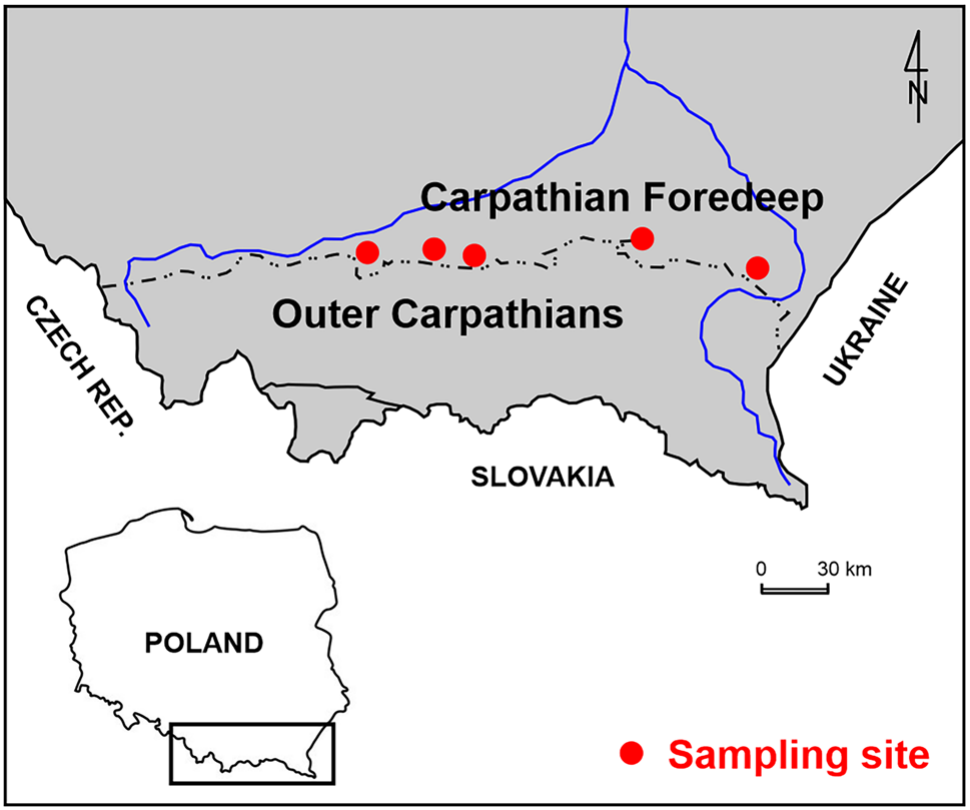
Location of the sampling area.

The soils used for the investigation were collected from the area that lies on the border of two polish geological units, i.e. the Carpathian Foredeep and the Outer Carpathians (see Fig. 2). On the basis of preliminary geological engineering analysis of the area, most of the sediments are Quaternary. In turn, the Miocene formations are the immediate bedrock of the Quaternary sediments. The samples were collected from the pseudo-loess cover. The core samples used for the laboratory investigation were taken from a depth of 1.65–7.85 m. The cover is a typical example of the subsoil representing the area of interest. The accumulation of a substantial part of it is associated with the period of the northern Polish glaciations. The acquired soil material is mixtures of silt, clay and sand fractions in different proportions, mainly loams, silty loams and sandy loams with various interlayers, inserts, and smaller or larger clusters that differ in grain-size distribution, colour, water content and consistency state. The separated lithological-genetic types of soils, despite the general similarity in their formation, are more or less heterogeneous. When considering soil behaviour, these soils should be treated as "intermediate soils", which are neither clay-like nor sand-like.

### Testing procedures

#### Index properties and classification of soils

Comprehensive index tests were carried out to allege the soil identification, description and classification. These tests included determining natural water content, consistency limits, specific gravity, bulk density and unit weight. The gravimetric method with oven drying was used to measure the natural water content (w_n_) as per ISO/TS 17,892-1^55^. The liquid limit (LL) and plastic limit (PL) were determined according to ISO/TS 17,892-6^56^ and CEN ISO/TS 17,892-12^57^, respectively. Using the PL and LL values, the plasticity index (PI = LL-PL) (%) was calculated, which allowed for utilising Casagrande’s chart for classification purposes. The particle-size distribution was identified prior to ISO-TS 17,892-4^58^, and the specific gravity tests were performed following the procedure described in ISO-TS 17,892-3^59^. It is worth noticing that organic matter in the tested soils was not found during the laboratory investigation. In the work presented herein, the soils were classified based on the Atterberg limits combined with criteria for delimiting clays, clayey soils and less plastic silty soils given by Moreno-Maroto^60^.

#### Consolidation tests

The compressibility characteristics of soils utilised in the study were determined by means of a conventional incremental loading oedometer test in accordance with the requirements of the ISO/TS 17,892-5^61^. The tests were performed on specimens placed in the consolidation ring with a size of 60 mm diameter and 20 mm thickness. The internal sides of the ring were lubricated with silicone grease to minimize side friction. Filter papers and porous stones covered each specimen's top and bottom faces. Testing soils were subjected to a loading–unloading path, and each load was kept for one day (24 h). The following loading scheme was executed: 12.5 kPa → 25 kPa → 50 kPa → 100 kPa → 200 kPa → 400 kPa → 800 kPa ← 12.5 kPa. The specimen deformation was expressed as relative compression (settlement) for each load increment, and the corresponding time was noted.

Interpretation of the compression curves to determine σ′_xy_ was performed using an open-source *pySigmaP* software^20^. This made it possible to remove the interpreter's judgment when choosing inflection points or points of maximum curvature in the e − log(σ′_v_) space, necessary for methods belonging to the semi-logarithmic group. The *pySigmaP*, developed in *Python 3*, is designated to identify the yielding of soils by incremental loading oedometer test. This software enables to determination the σ′_xy_ of the soils using nine different methods presented above. The *pySigmaP* package is divided into six modules: *data.py*, *casagrande.py*, *energy.py*, *bilog.py*, *pachecosilva.py*, and *boone.py*. The *data.py* module contains the class to load and manage the data of the compressibility soil response. The remaining modules contain the classes and methods to determine σ′_xy_. The *pySigmaP* provides a more objective and straightforward analysis to estimate σ′_xy_. The undeniable advantage of the software used is its calculations are guided by analytical and numerical methods minimising bias by the scale dependency, analyst visual skills, engineering judgment, and experience.

## Results and discussion

### Index properties and soil classification

**Figure 3:**
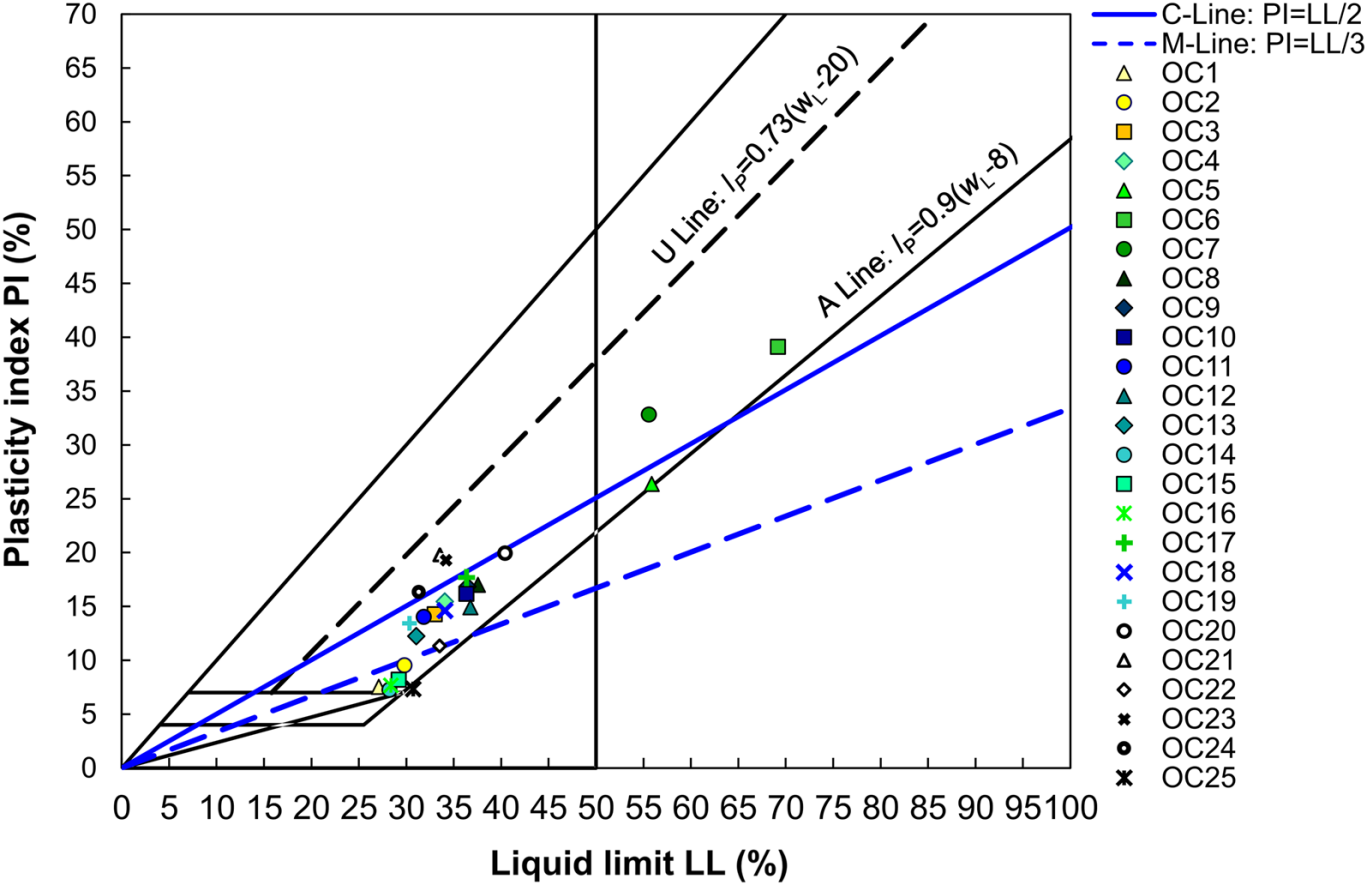
Classification of the soils used in the present study.

Table 1 presents the index and selected physical properties of the tested soils. It may be seen that the values of natural water content ranged from 13 to 35%, LL from 27 to 69%, PI from 7 to 39% and the percent clay size fraction from 11 to 35%. Based on the values of PI and LL and the boundaries established by Moreno-Maroto, most of the tested soils are located between the C and M lines on the plasticity diagram (see Fig. 3). This indicates moderately or slightly clayey soils. The following 5 points are located above C-line, which shows the clay’s limit. The remaining 4 points are below the M-line, which points to less-plastic silt–clay soils and mixtures of various fractions with the dominance of the silt fraction. As shown in Fig. 3, the classic Casagrande’s A-line that separates clays from silts leads to utterly different classification results. Considering the visual inspection of the soils, their grain-size distribution and the values of inherent parameters, such as PL and LL, it should be stated that in most cases, we are not dealing with clays but with soils containing clayey fraction that only modifies engineering properties of that soils.

**Table 1:** Physical parameters of intact intermediate soils utilised in the present study.

Sample number	Sampling depth	ρ (g/cm^3^)	G_s_ (-)	ρ_d_ (g/cm^3^)	e_0_ (-)	w (%)	PL (%)	LL (%)	PI (%)	LI (%)	F_Cl_ (%)	F_Si_ (%)	F_Sa_ (%)
OC1	7,35	1,98	2,7	1,63	0,656	19,01	19,57	27,10	7,54	0,02	15	79	6
OC2	7,55	1,93	2,68	1,56	0,717	17,17	20,25	29,81	9,56	0,02	17	76	7
OC3	8,25	2,00	2,68	1,66	0,614	20,33	18,72	33,00	14,29	0,11	21	68	11
OC4	1,65	1,98	2,71	1,67	0,622	19,56	18,59	34,05	15,47	-0,02	20	38	42
OC5	8,15	1,83	2,74	1,53	0,790	23,14	29,48	55,85	26,37	0,01	29	59	12
OC6	2,35	1,98	2,73	1,57	0,738	35,30	30,06	69,16	39,11	0,13	35	53	12
OC7	1,45	1,96	2,67	1,82	0,463	24,20	22,74	55,57	32,84	0,04	27	44	29
OC8	2,85	2,04	2,67	1,59	0,679	27,94	20,53	37,54	17,01	0,44	20	74	6
OC9	2,25	1,98	2,65	1,59	0,666	24,31	19,60	36,44	16,85	0,28	20	72	8
OC10	3,66	1,86	2,63	1,50	0,753	26,68	20,15	36,32	16,18	0,40	14	53	32
OC11	4,59	2,11	2,65	1,83	0,448	20,53	17,79	31,84	14,05	0,20	17	46	37
OC12	3,40	1,91	2,66	1,61	0,652	24,87	21,82	36,74	14,92	0,20	14	54	32
OC13	8,62	2,05	2,65	1,68	0,577	24,07	18,80	31,05	12,25	0,43	13	77	10
OC14	3,10	1,90	2,65	1,56	0,698	22,53	20,96	28,22	7,27	0,03	18	63	19
OC15	7,49	2,04	2,63	1,73	0,520	22,72	20,98	29,18	8,21	0,21	16	72	12
OC16	5,25	1,86	2,66	1,51	0,761	19,34	20,70	28,36	7,67	-0,18	16	71	13
OC17	8,67	2,07	2,64	1,76	0,500	20,83	18,63	36,34	17,71	0,07	17	64	19
OC18	6,35	1,94	2,66	1,61	0,652	22,17	19,44	34,06	14,62	0,16	17	72	11
OC19	7,27	2,03	2,67	1,73	0,543	21,38	16,90	30,33	13,44	0,33	17	63	20
OC20	5,32	2,04	2,69	1,71	0,573	21,37	20,47	40,40	19,94	0,05	21	65	14
OC21	6,25	2,66	2,15	1,88	0,414	14,46	13,73	33,54	19,81	0,02	17	42	40
OC22	7,43	1,99	2,72	1,61	0,689	22,81	22,16	33,50	11,35	0,06	17	42	40
OC23	4,10	2,05	2,69	1,77	0,519	16,47	14,91	34,20	19,29	0,12	18	40	42
OC24	7,20	2,19	2,70	1,95	0,384	13,50	15,02	31,34	16,33	-0,09	15	42	43
OC25	8,56	2,02	2,68	1,64	0,634	24,10	23,38	30,73	7,35	-0,3	11	55	33

F_Cl_ is the clay fraction, F_Si_ is the silt fraction, F_Sa_ is the sand fraction.

Based on the magnitudes of PI, testing soils, except for three samples, are identified as medium cohesive soils (PI = 10–20%). The remaining samples are medium/high cohesive soils (one sample with PI = 20–30%) and high-cohesive soils (two samples with PI = 30–40%). The soil’s plasticity was assessed based on the LL values. Seventeen out of 25 samples showed low plasticity (LL = 20–35%), another five samples indicated medium plasticity (LL = 35–50%), and the rest three samples showed high plasticity (LL > 50%).

### Compression of the soils

**Figure 4:**
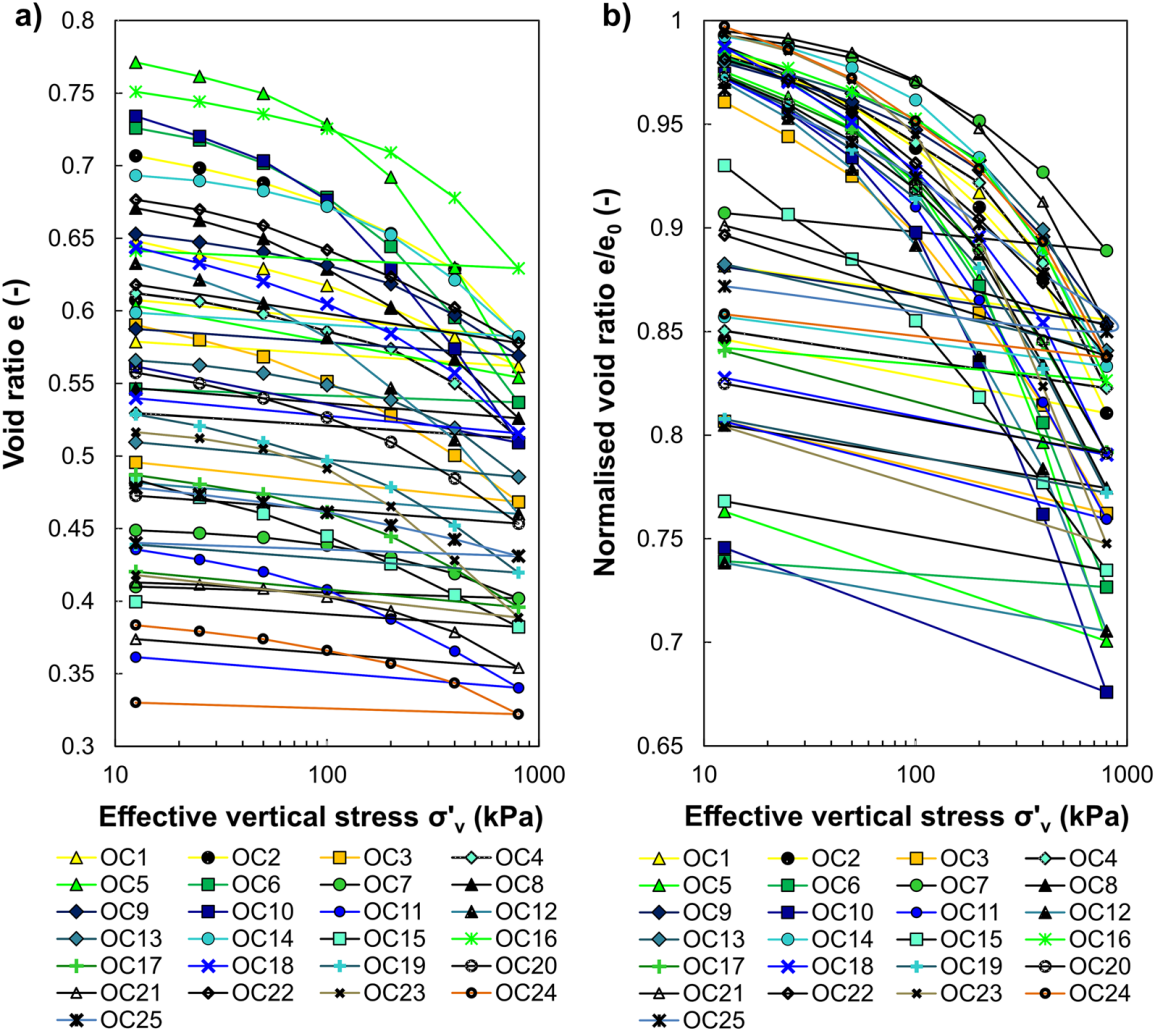
Soil compression curves in a semi-logarithmic plane: (**a**) void ratio vs effective vertical stress, (**b**) normalised void ratio vs effective vertical stress.

The semi-logarithmic plots of void ratio vs effective vertical stress and normalised void ratio vs effective vertical stress for 25 intact soil samples are shown in Fig. 4a,b. As can be seen, most of the e–log σ′_v_ relationships are initially curved concave downward. At the high values of the effective vertical stress, all exhibit well-defined straight lines or slightly pronounced concave upward course. The slopes of these lines are further utilised to derive the soil’s compression index (C_c_).

**Figure 5:**
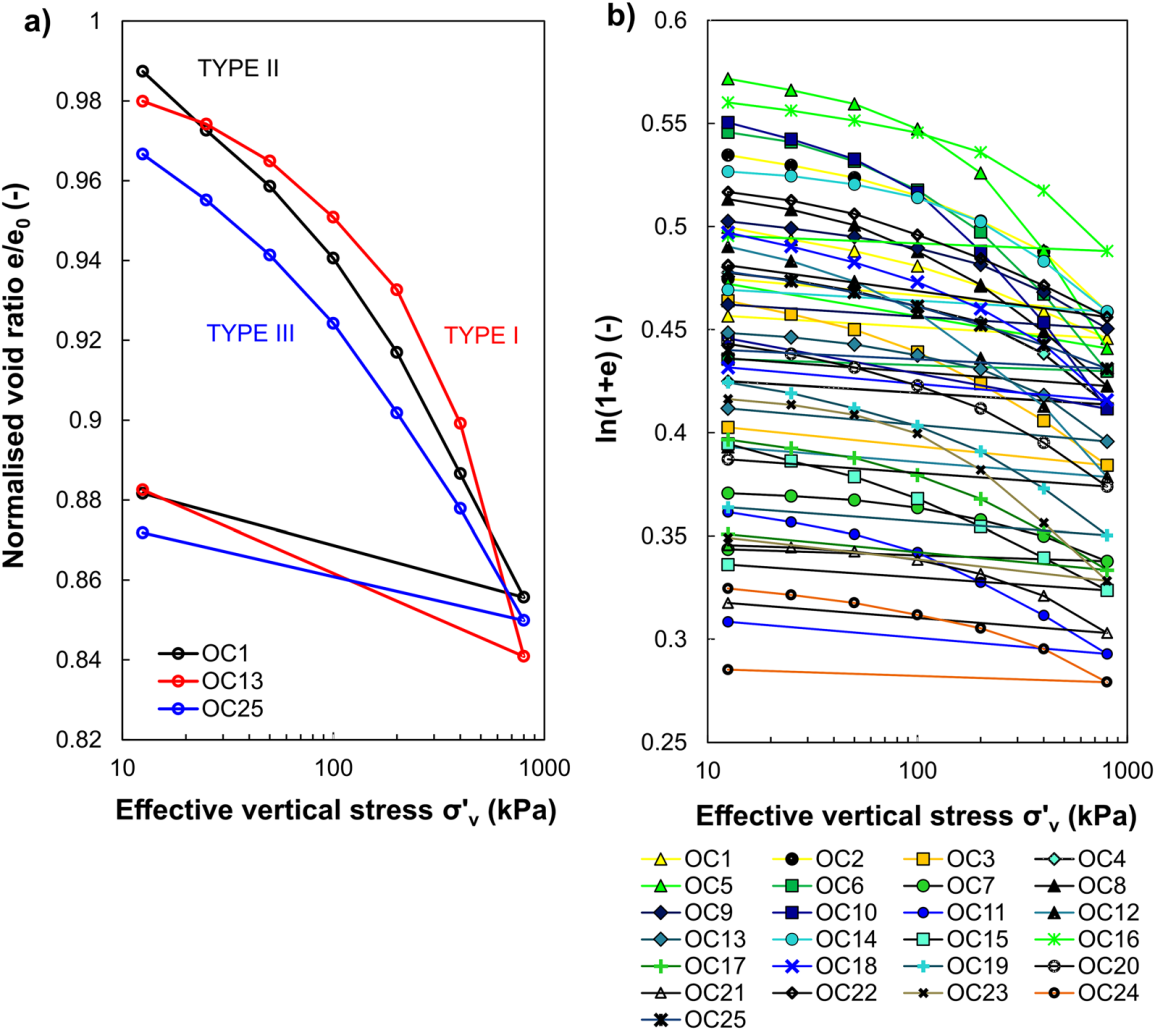
Soil compression behaviour: (**a**) types of compression curve in a normalised void ratio versus effective vertical stress space, (**b**) compression data plotted in a bi-logarithmic plane.

Inspection of the curves reveals some slight differences in the shape of their initial portions. Thus, the three patterns differ in the initial compression rate. The first case (type I) concerns mild changes in the parabolic shape of the curve with a not very clearly marked breakdown corresponding to a shift in soil behaviour (see soil OC13 in Fig. 5a). Therefore, the e–log σ′_v_ relationship is curved and concaves downwards throughout the stress ranges. This type is most common for the investigated soils (17 samples). It complies with the reported compression characteristics of compacted soils, soils with lower initial water content than their LL value^62^ or less-plastic soils. The second type of curve (type II) is similar in shape to the first one, except for the initial segment, which is a distinct rectilinear with a constant slope (see soil OC1 in Fig. 5a). This commonly may occur due to the significant soil disturbances created during sampling, transport and laboratory preparation/cutting of the specimen before the test. Compressibility curves classified as type II (i.e. OC1, OC15, OC16) in the plasticity diagram were located below the M-line, representing less-plastic silt-like soils. Finally, the initial curvature is flattened in the third case (type III), and the middle part of compression curve is convex upward (see soil OC25 in Fig. 5a). It should be noted that these differences are more visible when the vertical scale of the chart is reduced. Taking into account that the differences in the initial courses were minor and the middle and final sections of the compressibility curve are usually used for interpretation, the presented division into groups should be treated conventionally.

The compression behaviour of various fine-grained soils can be well represented by the two or three straight lines in the plot of ln(1 + e) against log σ′_v_ or log(1 + e) against log σ′_v_^63–65^. These lines are used to represent pre-yield, transitional, and post-yield compression behaviour^66^. As indicated by Hong et al.^66^, the small-compressibility refers to the pre-yield regime (overconsolidated region, OC), where changes in the soil structure are relatively small up to the σ′_xy_. The so-called "transitional regime" explained by transitional stress and gradual destructuration of soil structure can be observed in some soils. In turn, in the post-yield regime (normally-consolidated region, NC), the soils experience the most remarkable changes in compressibility. Figure 5b presents oedometric inverse S-shaped compression data replotted in the graph of ln(1 + e) vs log σ′_v_ for the investigated soils. It is observed that the shape of all analysed compression curves can be imitated by the two straight lines. Thus, the soil's compression follows bi-linear behaviour.

**Figure 6:**
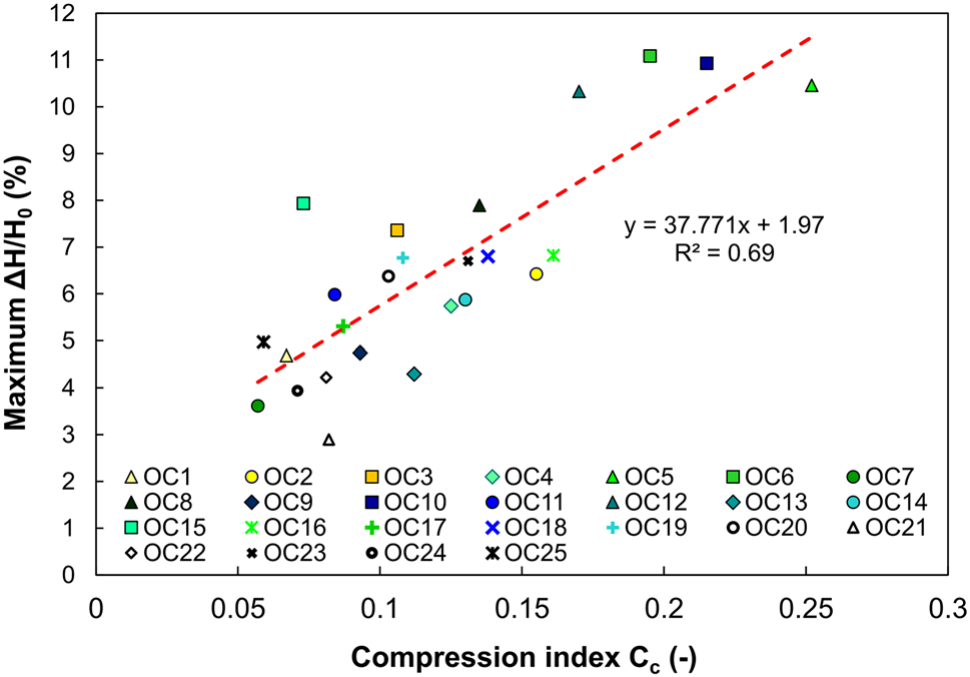
Relationship between compression index (C_c_) and maximum strain (ΔH/H_0_).

Table 2 listed compression parameters for the tested soils. The values of the calculated C_c_ for the stresses between 400 and 800 kPa were in the range of 0.057–0.252. In general, it can be stated that the higher the C_c_ value, the greater the maximum straining of the sample, as depicted in Fig. 6. It is common practice to correlate C_c_ with the Atterberg limits of the soil^67–69^ the water content, the clay fraction CF (%) or the organic matter content. Due to the origin of the soil from different locations and the adopted utilitarian goals of the article, a detailed description of these relationships was omitted because the primary intention of the undertaken research is a statistical assessment of the methods for determining σ′_xy_.

**Table 2:** Compression parameters of analysed soils.

Sample number	Sampling depth	C_c_	C_r_
OC1	7,35	0,067	0,009
OC2	7,55	0,155	0,014
OC3	8,25	0,106	0,015
OC4	1,65	0,125	0,010
OC5	8,15	0,252	0,027
OC6	2,35	0,195	0,005
OC7	1,45	0,057	0,005
OC8	2,85	0,135	0,011
OC9	2,25	0,093	0,010
OC10	3,66	0,215	0,029
OC11	4,59	0,084	0,012
OC12	3,40	0,170	0,012
OC13	8,62	0,112	0,013
OC14	3,10	0,130	0,009
OC15	7,49	0,073	0,010
OC16	5,25	0,161	0,007
OC17	8,67	0,087	0,014
OC18	6,35	0,138	0,013
OC19	7,27	0,108	0,011
OC20	5,32	0,103	0,011
OC21	6,25	0,082	0,011
OC22	7,43	0,081	0,022
OC23	4,10	0,131	0,016
OC24	7,20	0,071	0,004
OC25	8,56	0,059	0,008

### Determining the effective vertical yield stress σ′_xy_

**Figure 7:**
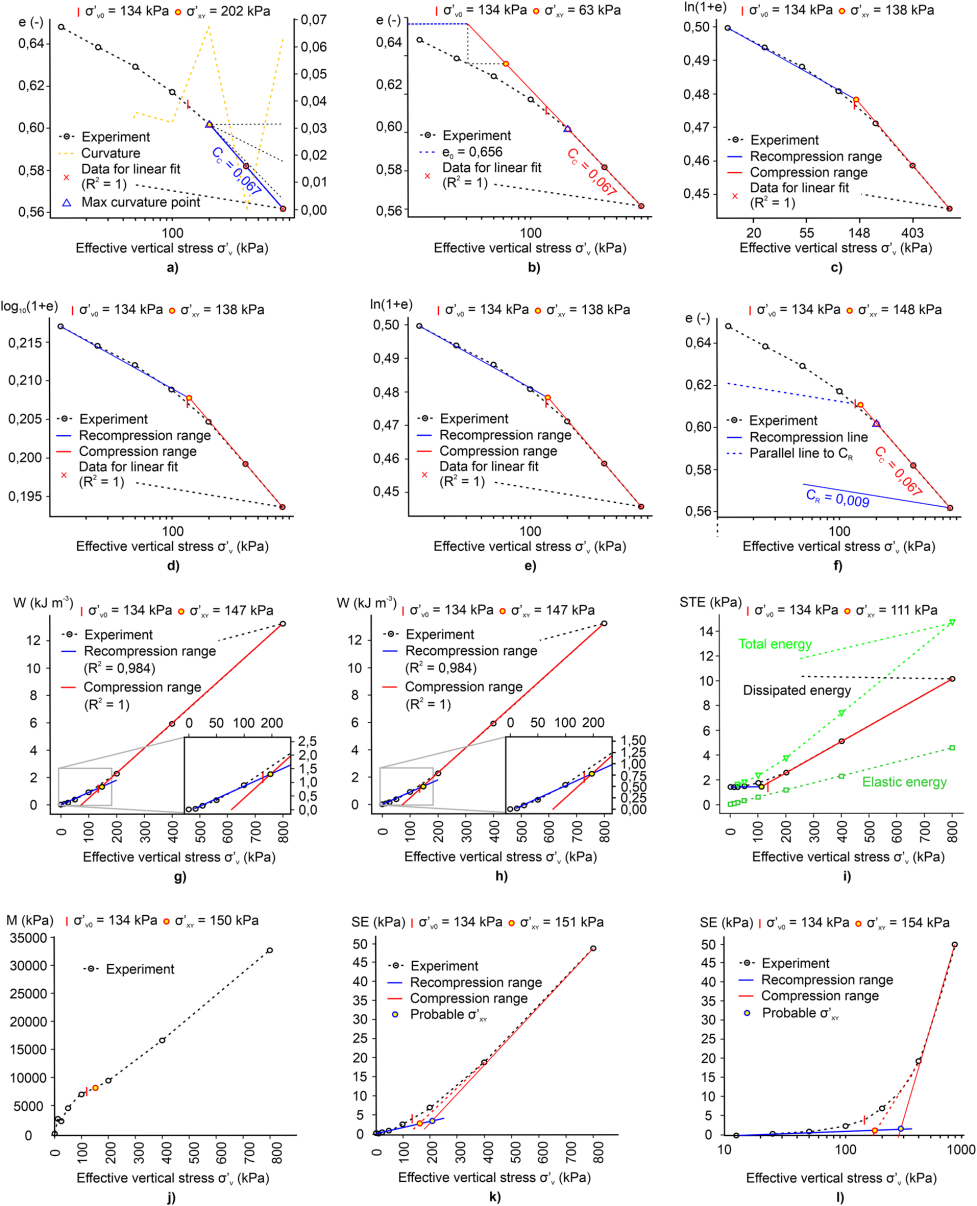
Exemplary plots for interpreting oedometer data of sample OC1 by the semi-logarithmic methods, bi-logarithmic methods and the work approach: (**a**) CM, (**b**) PSM, (**c**) BTM, (**d**) OIM, (**e**) ONM, (**f**) BM, (**g**) WM, (**h**) WPUVSM, (**i**) DSEM, (**j**) JM, (**k**) SEM, (**l**) SLSEM.

In order to determine σ′_xy_, the data from oedometric tests performed on intermediate soils were converted and replotted on the appropriate diagrams. The typical results of interpretation using the *pySigmaP* software for soil sample OC1 are shown in Fig. 7. The first step after loading the data was to plot the compression curves and determine their basic parameters, such as C_c_ and C_r_. For the CM, a cubic spline function was used to determine the maximum point of curvature on the compression curve. As can be seen, this value was established as 202 kPa (see Fig. 7a). The use of the *pySigmaP* excludes the subjective interpretation of this point because the mathematical formulation for the curvature of the soil compression response as a function of the stress level defines it. Figure 7b shows the plot for the PSM. After determining the initial e_0_ value, the elongation procedures of the linear part of the compression curve were performed. For the discussed soil sample OC1, the value of σ′_xy_ determined by the PSM was 63 kPa. The large scatter of values between these two semi-logarithmic methods was most likely the result of the behaviour of the soil sample, which, when subjected to loads, exhibited a flat-shaped course in the initial part of the curve^70^. The result of the reduced initial compressibility of the soil is the low value of σ′_xy_ determined by the PCM, which is principally based on the graphical construction, taking into account the initial course of the compression curve. This problem appears practically only in the case of PCM.

As can be traced in Figs. 7c,d,e, data in bi-logarithmic diagrams are ideally represented by two straight lines, and their intersection indicates a change in soil behaviour from smaller to larger compressibility. Irrespective of the relationship used, the same σ′_xy_ value of 138 kPa was obtained in these methods. Figure 7f shows the compressibility diagram for the BM. The value of σ′_xy_, in this case, was 104 kPa and was smaller than those determined by the CM and bi-logarithmic methods.

Figures 7g,h present the results of two methods based on the work approach for determining σ′_xy_. Since both methods differ only in the mathematical formulation of the work per unit volume for each load increment, the determined values of σ′_xy_ for the analysed soil samples are the same. For the discussed soil sample, the σ′_xy_ was established as 147 kPa. This value was practically identical to the σ′_xy_ obtained with BM and close to the values determined by bi-logarithmic methods. Figure 7i depicts the diagram for the DSEM. Since this method uses dissipated strain energy that considers both elastic and plastic deformation, the obtained value of σ′_xy_ = 111 kPa was lower than those determined by WM and WPUVSM. The obtained value was also smaller when compared with other methods other than BM and PSM.

Figures 7j,k,l present the plots for determining σ′_xy_ by the JM, SEM and SLSEM. During the interpretation of the compression curve for the OC1 sample as well as for some other samples, problems with the application of these methods were encountered. In the case of JM, it was not always possible to locate the point where the curve bends downwards (i.e. method requires the judgement and experience of the interpreter). In addition, some relationships between M and σ′_v_ showed a practically increasing linear course. In the case of SEM and SLSEM, choosing a rectilinear segment and drawing tangents was problematic (see Fig. 7k,l). The results obtained with these two methods are ambiguous and difficult or even impossible to apply for many of the analysed compressibility curves. Moreover, with strict adherence to the guidelines provided by the authors of the SLSEM method, the determined σ′_xy_ values were significantly inflated compared to other methods.

## Methods comparison study

In this subsection, a method comparison study is attempted. Due to the lack of a reference method admitted according to current practice as ‘the best” and most reliable, the centre of attention was on assessing the degree of agreement between different methods and the precision of the methods. Method comparison is the statistical process by which error components are recognised and characterised. Thus, errors within acceptable limits may indicate the adequacy of the given method and point to its reliability. In this work two-step approach for comparison purposes was applied. At first, the scatter plots for analysing the comparative results have been constructed. The extreme outliers have been identified and, in some cases, excluded during the trimming of the data. The slope and intercept of the trimmed data using a regression procedure were also determined to consider the convergent validity of compared methods. In addition, the variance of the slope from the identity line that formed a 45° angle with the abscissa has been used to indicate a bias. The strength of the linear association between the two compared methods was evaluated by the Pearson product-moment correlation coefficient (r). In this way, the r-value helps establish convergent validity showing whether two methods are related to each other. Note that The calculated r-values were not used to measure the accuracy of methods or to assess the agreement between them.

In the case of compression laboratory data, neither provides an unequivocally correct determination of σ′_xy_ when comparing the two methods. Therefore, assessing the degree of agreement between these methods might be helpful. To evaluate the degree of agreement between the methods, the common practice is to study the mean difference between them and to establish limits of the agreement^71–74^. The limit of agreement is referred to the confidence interval that represents the range of values in which agreement between methods lies for approximately 95% of the sample. A difference plot and analysis of differences may be used to compare two “new” methods or one method against a reference one^75^. The second part of the analysis was based on establishing the statistical limits by using the mean and the standard deviation (SD) of the difference (indice of precision). In the difference plot, the Y-axis expresses the difference between the two paired values, and the X-axis represents the average. This allows for checking the assumption of normality of differences.

### Association between the methods

**Figure 8:**
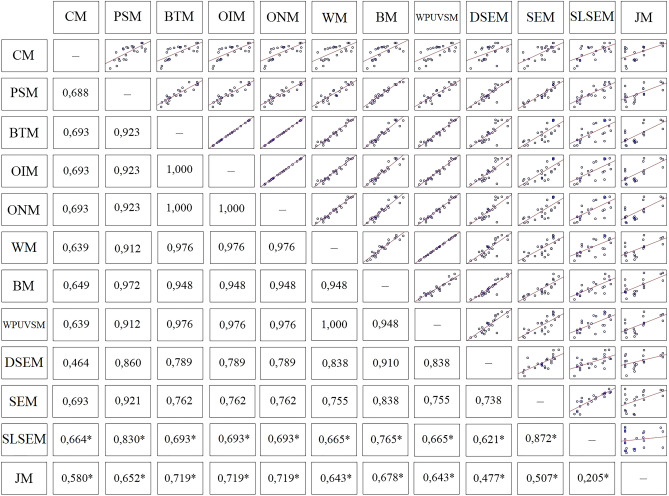
Correlation matrix for convergent validity analysis (Note: *the correlations were performed on a reduced number of samples due to outliers).

A cross-correlation analysis was performed for the collected values of σ′_xy_ to assess whether the individual values determined by twelve different methods are statistically significantly related. Figure 8 presents the results of this analysis as a correlation matrix with scatter plots and computed r-values for the probability value *p* < 0.05. Due to the same procedure and nature of determining σ′_xy_ for three bi-logarithmic methods, a complete convergence of σ′_xy_ values was observed. The same relation was observed for the WM and WPUVSM.

The CM showed a low correlation with other methods of determining σ′_xy_. The best link was found with the bi-logarithmic methods and with the SEM, for which the r = 0.693. In the same group of semi-logarithmic methods, σ′_xy_ using the CM correlated with those determined using the PSM with r = 0.688 and the BM with r = 0.649. The values of σ′_xy_ by the CM are associated with the SLSEM with r = 0.664 and the WM and WPUVSM with r = 0.639. The lowest correlations were obtained for the CM and DSEM, where r = 0.464 and the CM and the JM with r = 0.580. These results indicated that those correlations cannot be considered as significant. Many different factors can cause the low level of correlation of the CM with other methods. It is believed that most important is the deformation properties of the tested soil samples, for which the initial sections of the compression curve exhibited a relatively flat geometry and the accompanying scale effect. However, the subjectivity of finding the inflection point on the curve can be ruled out as the software determines it. It is also worth noticing that the CM gives higher values of σ′_xy_ for the vast majority of samples compared to other methods, which also affects the value of r. In the group of semi-logarithmic methods, the PSM and the BM showed a high correlation with r = 0.972. This may be due to the similar methodology for determining σ′_xy_. The PSM is also well correlated with the SEM, for which r = 0.921, and the bi-logarithmic methods, for which the r was 0.923.

Satisfactory results were also obtained between the bi-logarithmic methods and the work methods. Concerning the WM and WPUVSM, the r values were the highest among all comparisons and amounted to 0.976. With the DSEM, this value was 0.789. Bi-logarithmic methods correlated equally well with semi-logarithmic PSM with r = 0.923 and Boone with r = 0.948. All determinations of σ′_xy_ made with DSEM correlated moderately with the other methods. The r values for correlations between this method and the BM, the PSM and the WM and WPUVSM were respectively 0.910, 0.860 and 0.838. The values of σ′_xy_ obtained using the SEM correlated moderately with those from other methods, preferably with the PSM, for which the r was 0.921, and with the BM (r = 0.838). The association of the SEM and the bi-logarithmic methods, the WM together with WPUVSM and the DSEM were slightly weaker, with r amounting to 0.762, 0.755 and 0.738, respectively. The weakest correlations occurred between the SEM and CM with r = 0.693 and the JM with r = 0.507.

The SLSEM correlated best with the SEM with r = 0.872 and PSM with r = 0.830 and was at the same moderate level of correlation. The BM achieved a value of r = 0.765, the WM and WPUVSM 0.755, the bi-logarithmic methods 0.693, and the DSEM 0.621. The SLSEM and the JM exhibited the weakest correlation with r = 0.352. Like the CM, the SLSEM receives higher values of σ′_xy_ than other methods. The JM correlates best with the SEM, for which the r was 0.711. In turn, the correlation considering the JM, with r = 0.205, was the weakest. On the other hand, the JM correlated best with bi-logarithmic methods. The correlation value between them was determined at the level of 0.719.

To conclude, the results of the correlational analysis revealed that the bi-logarithmic methods correlated best with the other methods of determining σ′_xy_. The strongest correlations were observed between the bi-logarithmic methods and the methods based on work approach, i.e. WM and WPUVSM (r = 0.976); slightly worse correlations were archived with the semi-logarithmic methods of BM (r = 0.948) and PSM (r = 0.923) and the DSEM (r = 0.789), SEM (r = 0.762), and JM (r = 0.719).

### Agreement between the methods

The difference charts were used to assess the compatibility of particular methods with each other. In the work presented herein, the primary purpose of the analysis was to assess the differences between the results obtained from different methods for determining σ′_xy_. According to the division presented earlier, the methods have been assigned to individual groups. Therefore, one method was selected from each group, and its results were compared with the others. The following three cases were taken for comparison. From semi-logarithmic methods, CM, bi-logarithmic BTM and methods based on the work approach DSEM were selected.

**Figure 9:**
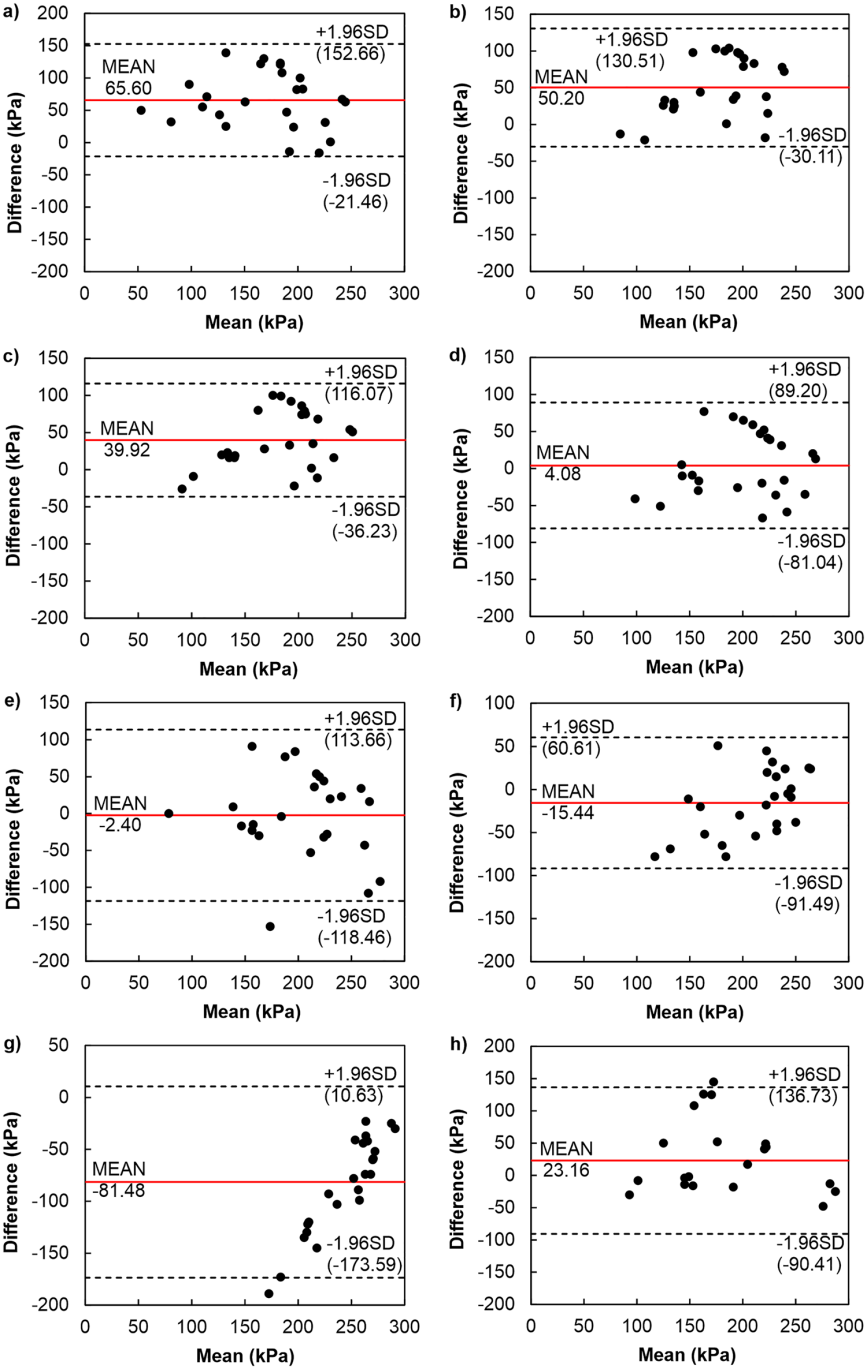
Difference plots comparing semi-logarithmic method (CM) with other methods: (**a**) PSM, (**b**) BM, (**c**) BTM, OIM and ONM, (**d**) WM and WPUWSM, (**e**) DSEM, (**f**) SEM, (**g**) SLSEM, (**h**) JM.

For the group of semi-logarithmic methods, an investigation was carried out between the values of σ′_xy_ determined by the CM, which is a standard approach recommended, for instance, in the EC-7 code, and methods described earlier (see Fig. 1). Figure 9 shows the difference plots comparing estimated values of σ′_xy_ by CM with those using other methods. The analysis revealed that the σ′_xy_ values determined by CM are superior to the other semi-logarithmic methods (i.e. PSM—by 65.6 kPa on average, BM—by 50.2 kPa on average, and bi-logarithmic methods—by 39.92 kPa on average). The ranges of compliance intervals for semi-logarithmic methods are 174.13 kPa and 160.62 kPa, respectively, and 152.30 kPa for bi-logarithmic methods. The difference plots indicated that the minor differences in values from the CM are achieved by methods based on the work principle. Determinations of σ′_xy_ by the CM concerning the WM and the WPUVSM gave higher results on average by about 4 kPa (span of the compatibility interval equals 170.24 kPa) and compared to the DSEM lower by 2.4 kPa on average (span of the compatibility interval equals to 232.12 kPa).

The uniform nature of the dispersion for these methods proves the lack of correlation between the average values of the repeated determinations by the two methods and the differences between them. All plot points lay within the agreement area, meaning all differences that appeared were due to measurement error. The remaining three methods, SEM, SLSEM and JM, gave σ′_xy_ values higher than those from the CM by an average of 15.44 kPa, 81.48 kPa, and 23.16 kPa with extensive agreement intervals of 152.1 kPa, 184.22 kPa, 227.14 kPa, respectively. As can be seen, similar results were obtained in two cases; on average, the DSEM determined 2.4 units more than the CM, and on average, the WM, together with WPUWSM, specified 4 units less than the CM.

**Figure 10:**
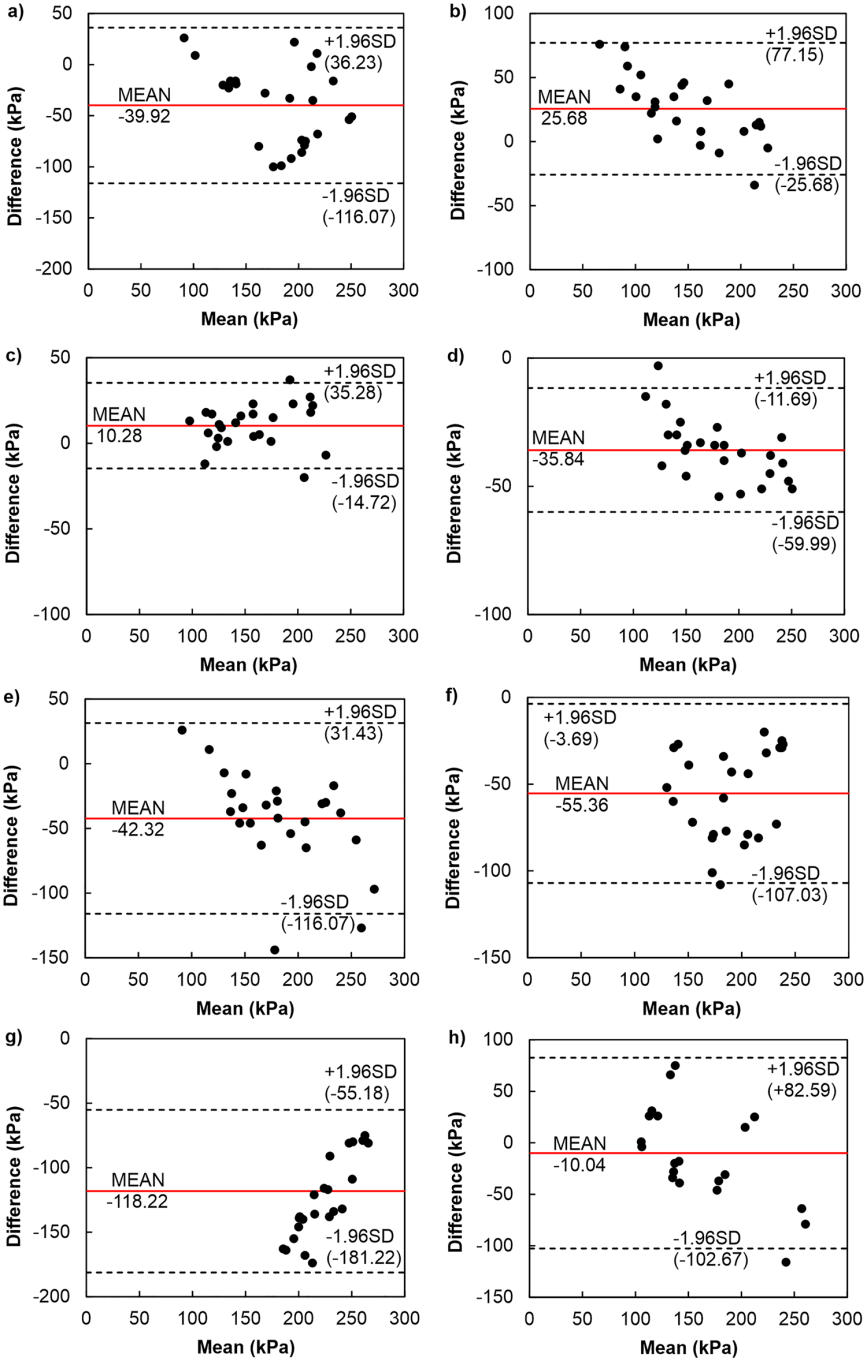
Difference plots comparing bi-logarithmic methods (BTM, OIM, ONM) with other methods: (**a**) CM, (**b**) PSM, (**c**) BM, (**d**) WM and WPUVSM, (**e**) DSEM, (**f**) SEM, (**g**) SLSEM, (**h**) JM.

The bi-logarithmic methods showed a better agreement between the σ′_xy_ values compared to other methods (see Fig. 10). The analysis revealed that the σ′_xy_ data originated from bi-logarithmic methods were higher than the PSM values by an average of 25.68 kPa at the span of the agreement interval of 102.95 kPa, and against the BM values by 10.28 kPa at the span of the agreement interval of 50 kPa. In the case of one soil sample tested with the PSM and two with the BM, the points were outside the agreement area, proving that the differences in these specific cases may result from a random factor. The values of σ′_xy_ determined by the bi-logarithmic methods were lower than those specified by work methods. For the WM and WPUVSM, the values were lower by an average of 35.84 kPa with a span of the compliance interval of 71.68 kPa, and for the DSEM by 42.32 kPa on average with a span of a compliance interval of 158.39 kPa. Of the assays described, four samples fell outside the agreement range. The values of σ′_xy_ determined by bi-logarithmic methods were also compared to the SEM, SLSEM, and JM and were lower by an average of 55.36 kPa, 118.20 kPa, 10.4 kPa with the range spans of 110.72 kPa, 236.40 kPa and 185.26 kPa, respectively. To conclude, similar results were obtained in two cases; on average, the BM determined 10 units less than the bi-logarithmic methods, and on average, the JM, specified 10 units more than the bi-logarithmic methods. It also should be noted that the span between BTM, OIM, and ONM values and those of BM was the lowest in all considered cases.

**Figure 11:**
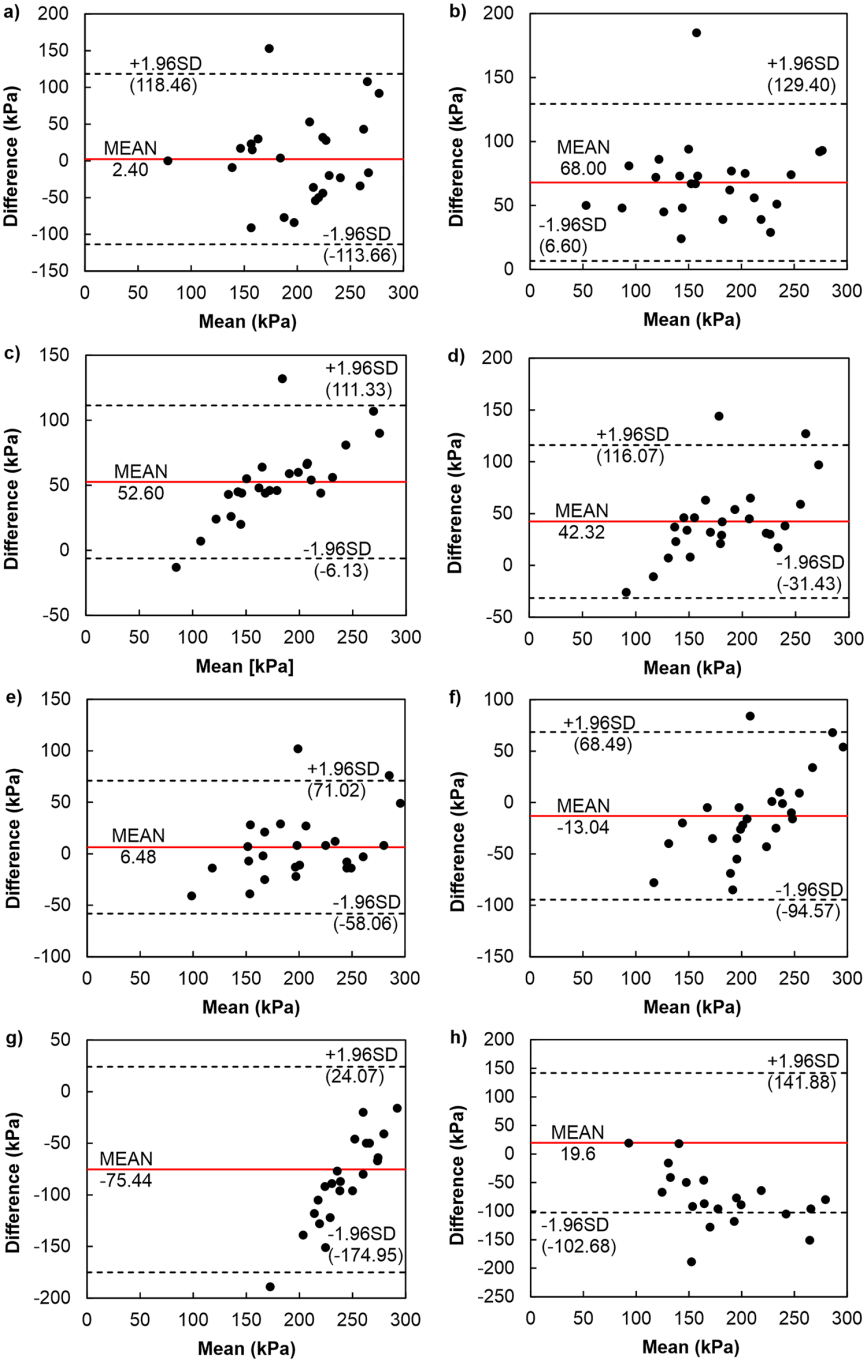
Difference plots comparing DSEM with other methods: (**a**) CM, (**b**) PSM, (**c**) BM, (**d**) BTM, OIM and ONM, (**e**) WM and WPUVSM, (**f**) SEM, (**g**) SLSEM, (**h**) JM.

The last group of methods for determining σ′_xy_, for which the difference analysis has been carried out, was the methods based on work from which the DSEM was selected (see Fig. 11), giving the best convergence of results with the CM. This method showed higher values of σ′_xy_ than semi-logarithmic methods (PSM—on average by 68 kPa, BM—on average by 52.6 kPa) and bi-logarithmic methods (on average by 43.32 kPa). The ranges of agreement intervals for semi-logarithmic methods were 136 kPa and 117.47 kPa, respectively, and for bi-logarithmic methods 147.49 kPa. In this group, the best convergence of σ′_xy_ results was achieved by the DSEM compared to the WM and WPUVSM. It gave higher parameter values on average by 6.48 kPa with a compliance interval of 129.09 kPa. The values of σ′_xy_ obtained by the DSEM compared to the SEM and SLSEM were lower by an average of 13.04 kPa and 75.44 kPa, with the agreement ranges being 163.06 kPa and 199.02 kPa, respectively.

Concerning the JM, the values of σ′_xy_ were higher by an average of 19.6 kPa with the span of the compliance interval of 244.56 kPa. For all analyses performed, only two determinations exceeded the designated agreement ranges. For the last group, similar results were obtained in two cases; on average, the CM determined 2.4 units more than DSEM, and on average, the WM and WPUVSM specified 6.48 units more than DSEM.

The conducted analyses allow us to conclude that the best convergence of the results of σ′_xy_ with respect to the CM was demonstrated by the DSEM. However, it should be emphasised that the commonly used CM is not the best reference. As shown by the previous considerations and analyses (e.g.^43,76^), this is not an exact method because it is associated with the so-called effect of scale and the subjectivity of finding the inflection point of the compressibility curve. This significantly impacts the repeatability of parameter determination, which is an essential but often neglected part of statistics in the study of compliance between methods. The method with more excellent repeatability is more precise. Suppose the measurements of one of the compared methods are not reproducible. In that case, its agreement with the other method will be low, as shown in the graphs (see Figs. 9, 10 and 11). If the repeatability of both methods is poor, then their agreement will be even lower. Consequently, when the reproducibility of the “old” method is poor, the compatibility of the ”new” one may be poor, even if the ”new” has high reproducibility.

### Final remarks

This paper elaborates on the incremental loading oedometer test results conducted on intermediate fine-grained soils. The 25 soil samples were one-dimensionally loaded to capture the compression behaviour and to determine effective vertical yield stress. Most of the void ratio vs effective vertical stress curves were described as initially curved concave downward with a more or less pronounced breakdown in their middle portion courses. The analysis identified three types of compressibility curve, differing slightly based on the initial compression rate. However, this division did not substantially affect the application of the methods for determing σ′_xy_. Successfully, the compression behaviour of this kind of soil has been represented by the two straight lines in the plot of ln(1 + e) against log σ′_v_.

In the case of interpreting the test results, not all methods were easy to apply, or there were problems with unequivocally determining the reliable value of σ′_xy_ on their basis. This mainly concerned SEM, SLSEM and JM. JM was the most difficult to use because there was practically no concavity observed in the plot of M against σ′_v_ associated with the σ′_xy_ for the investigated soils. Through the *pySigmaP* software used, judgment in identifying the maximum point of curvature as well as the dependency of analyst visual skills was eliminated. The following conclusions were drawn based on the findings concerning the comparative analysis of twelve methods presented in the previous subsections:

1. The CM and BM are the most time-consuming and complex in terms of graphical or analytical-graphical procedures, belonging to the group of semi-logarithmic methods. Based on mean difference analysis, the most similar values of σ′_xy_ to those given by CM were determined by methods based on the concept of work. The σ′_xy_ values by the WM and WPUVSM were, on average, 4 kPa lower than those from CM. In turn, the values derived from DSEM were, on average, 2.4 kPa higher than those obtained from CM. Since using CM involves considerable judgment during interpretation, a much more straightforward and firmly theoretical approach based on work is recommended.
2. Among the other semi-logarithmic methods, the σ′_xy_ values determined using PSM and BM differed from CM by 65 kPa and 50 kPa, respectively. Thus, the bias between PSM and BM amounted to 15 kPa.
3. Considering the bi-logarithmic methods (i.e. BTM, OIM and ONM), complete convergence of the determined σ′_xy_ values has been observed for all analysed samples. This is due to the same determining procedure and the lack of change in the course of the compressibility curve regardless of the log (1 + e) − log σ′_v_ or ln(1 + e)—log σ′_v_ graph used. The same situation pertained to methods based on the concept of work, i.e. WM and WPUVSM. However, it should be borne in mind that methods based on two logarithmic scales may hide the scatter of the recorded data during the test; therefore, it is suggested to concentrate the points on the curve or even smooth its course before applying them.
4. The convergent validity analysis demonstrated the highest positive association between bi-logarithmic methods and WPUVSM (i.e. r = 0.976) and bi-logarithmic methods and BM (i.e. r = 0.976), whereby the bias between them was about 36 kPa and 10 kPa, respectively. This means that regardless of the shape of the investigated compression curve, the type of soil or the sampling depth, if one has two related methods, the interpreter who scores, for instance, a high value of σ′_xy_ by one method should score high by the other as well.
5. Mean difference analysis using difference plots showed that relatively similar σ′_xy_ values were obtained using the following methods: CM against methods based on work approach (i.e. WM, WPUWSM, and DSEM) and bi-logarithmic methods against BM and JM. In this connection, at least three values of σ′_xy_ determined by different methods should be averaged and adopted as reliable. Considering the results of the analyses performed, WPUVSM, BM, and BTM can be used. However, it should be remembered that in most comparisons, there were extensive ranges of established agreement limits, which may obscure the unambiguous assessment of all methods. They are all based on more or less complex graphical procedures, and it is practically impossible to say which approach is correct.

## Data Availability

The datasets generated during and/or analysed during the current study are available from the corresponding author on reasonable request.
